# Approximation of the infection-age-structured SIR model by the conventional SIR model of infectious disease epidemiology

**DOI:** 10.3389/fepid.2024.1429034

**Published:** 2024-12-17

**Authors:** Ralph Brinks, Annika Hoyer

**Affiliations:** ^1^ Medical Biometry and Epidemiology, Faculty of Health/School of Medicine, Witten/Herdecke University, Witten, Germany; ^2^ Biostatistics and Medical Biometry, Medical School OWL, Bielefeld University, Bielefeld, Germany

**Keywords:** effective reproduction number, net reproduction number, influenza, SARS-CoV-2, Lexis diagram, Spanish flu

## Abstract

During the SARS-CoV-2 pandemic, the effective reproduction number (R-eff) has frequently been used to describe the course of the pandemic. Analytical properties of R-eff are rarely studied. We analytically examine how and under which conditions the conventional susceptible–infected–removed (SIR) model (without infection age) serves as an approximation to the infection-age-structured SIR model. Special emphasis is given to the role of R-eff, which is an implicit parameter in the infection-age-structured SIR model and an explicit parameter in the approximation. The analytical findings are illustrated by a simulation study about an hypothetical intervention during a SARS-CoV-2 outbreak and by historical data from an influenza outbreak in Prussian army camps in the region of Arnsberg (Germany), 1918–1919.

## 1 Introduction

The susceptible–infected–removed (SIR) model is a frequently used model in infectious disease epidemiology dating back at least to Kermack and McKendrick (1). In the SIR model, the population is partitioned into *susceptible*, *infected*, and *removed* (the initial letters of which give the model’s name “SIR”). To take into account varying transmissibility during the infectious period, sometimes a generalized conventional SIR model, known as the infection-age-structured SIR model, is considered. Both models are described by a set of differential equations. While the conventional SIR model is easy to understand and frequently used, the infection-age-structured SIR is slightly more complex. In this work, we seek for an approximation to simplify the differential equations of the infection-age-structured SIR model. In both models, conventional and infection-age-structured SIR, the *removed* state comprises people recovered and deceased from the *infected* state. The numbers of the people in the susceptible and the removed states at time t are denoted by S(t) and R(t), respectively.

The conventional SIR model is described in the next section. We start with the infection-age-structured SIR model, where the function i(t,τ) denotes the density of infected people at time t and duration τ since infection (i.e., the *infection age*). The number of infected at time t [I(t) ] is

(1) $$I(t)=\int_{0}^{\infty }i(t,\tau )\text{d}\tau .$$

The transmission rate of the infected people with infection age τ (i.e., the duration since infection) is β(t,τ) and the removal rate from the infectious stage is γ(τ). The rate γ comprises mortality as well as remission. According to Inaba (2), we can formulate the model equations for the infection-age-structured SIR model as follows:

(2) $$\frac{\text{d}S(t)}{\text{d}t}=-\lambda (t)S(t)$$

(3) $$\left(\frac{\partial }{\partial t}+\frac{\partial }{\partial \tau }\right)i(t,\tau )=-\gamma (\tau )i(t,\tau )$$

(4) $$\frac{\text{d}R(t)}{\text{d}t}=\int_{0}^{\infty }\gamma (\tau )i(t,\tau )$$

The incidence rate λ in Equation 2 is given by

(5) $$\lambda (t)=\int_{0}^{\infty }\beta (t,\tau )i(t,\tau )\text{d}\tau$$

which is usually called the *force of infection* (2).

Systems 2–4 are accompanied with the following initial and boundary conditions:

(6) $$S(0)=S_{0}$$

(7) i(t,0)=λ(t)S(t)

(8) $$i(0,\tau )=i_{0}(\tau )$$

(9) $$i(0,0)=S_{0}\int_{0}^{\infty }\beta (0,\tau )i_{0}(\tau )\text{d}\tau$$

where $S_{0}$ is assumed to be positive and $i_{0}$ is assumed to be non-negative and integrable. Apart from non-negativity, $i_{0}$ can have any distribution. For later use, we additionally assume that $i(t,\infty )\;:\;=\lim_{r\to \infty }i(t,\tau )=0$. Condition 9 is called *coupling equation* and guarantees that Systems 2–4 is well-defined [see Chen et al. (3) for details]. Note that Systems 2–4 are a generalization of the SEIR model (2), where SEIR means a model consisting of the states *susceptible, exposed, infected*, and *removed*. Detailed discussion of Equations 2–4 with initial Conditions 6–9 can be found in Inaba (2), such that we can be brief here.

Denoting the probability of still being infected at infection age τ with Γ(τ),

(10) $$\Gamma (\tau )\;:\;=\exp\left(-\int_{0}^{\tau }\gamma (\sigma )\text{d}\sigma \right)$$

the effective reproduction number R(t) is defined by (4, Equations 22 and 23)

(11) $$R(t)=S(t)\int_{0}^{\infty }\beta (t,\tau )\Gamma (\tau )\text{d}\tau$$

Note that the reproduction number R defined in Equation 11 is occasionally called *instantaneous* reproduction number (5).

A typical situation in infectious disease epidemiology is that the transmission rate β(t,τ) and the initial Conditions 6–9 are given. Then, Systems 2–4 are solved and the effective reproduction number R is calculated by Equation 11. In this way, R can be seen as an implicit or indirect parameter for the infection-age-structured SIR model, because R(t) can be calculated via Equation 11 *after* solving the governing Equations 2–4 with initial Conditions 6–9. In some situations, however, R(t) can be estimated more easily from population surveys than the transmission rate β(t,τ). Especially, in early phases of an outbreak of a new pathogen, the function β is frequently unknown. Then, the question arises if and how the infection-age-structured SIR model can be solved if the effective reproduction number R is given instead of β. In this case, we ask for a direct (or explicit) dependency of the differential equations on the parameter R.

## 2 Approximation of the age-structured SIR model by the conventional SIR model

In case the transmission rate β(t,τ) depends only on calendar time t, i.e., β(t,τ)=β(t), the force of infection can be written as

$$\lambda (t)\overset{5,7}{=}\beta (t)\;I(t)\overset{11}{=}\frac{R(t)\;I(t)}{S(t)\;\int_{0}^{\infty }\Gamma (\tau )\text{d}\tau }.$$

Then, Systems 2–4 become explicitly dependent on R. This means that for given R, the system can be numerically solved, for instance, by the algorithm described in the supplement to Brinks et al. (6). This is advantageous in situations when the effective reproduction number R is known while the transmission rate β is unknown. Note that there are a variety of methods for estimating R from a time series of numbers of incident cases, see, e.g., Fraser and Galvani (5) and Cori et al. (7). The question arises under which conditions Systems 2–4 can be approximated by the simpler conventional SIR model that explicitly depends on R. As shown at the beginning of this section, this is the case if the transmission rate β is independent from the infection age τ, i.e., β=β(t). For many diseases, however, there is a non-negligible dependency on the infection age τ, as for example, in SARS-CoV-2, see He et al. (8).

The conventional SIR model is a simpler model than the infection-age-structured SIR. The meaning of the variables is the same as above; however, the infection age τ is not considered. The governing equations of the conventional SIR model are as follows:

(12) $$\frac{\text{d}S(t)}{\text{d}t}=-\lambda (t)S(t)$$

(13) $$\frac{\text{d}I(t)}{\text{d}t}=\lambda (t)S(t)-r(t)I(t)$$

(14) $$\frac{\text{d}R(t)}{\text{d}t}=r(t)I(t)$$

To show similarities between the two SIR models, we start by applying Leibniz’s integral rule to Equation 1. The temporal derivative $\frac{\text{d}I}{\text{d}t}$ of the number of infected in Equation 1 can then be expressed as

(15) $$\begin{array}{rl} \frac{\text{d}I}{\text{d}t} & =\frac{\text{d}}{\text{d}t}\int_{0}^{\infty }i(t,\tau )\text{d}\tau =\int_{0}^{\infty }\frac{\partial }{\partial t}i(t,\tau )\text{d}\tau \\ & \overset{3}{=}-\int_{0}^{\infty }\gamma (\tau )i(t,\tau )\text{d}\tau -\int_{0}^{\infty }\frac{\partial }{\partial \tau }i(t,\tau )\text{d}\tau \\ & =-\int_{0}^{\infty }\gamma (\tau )i(t,\tau )\text{d}\tau -i(t,\infty )+i(t,0) \end{array}$$

As i(t,∞)=0 (by the assumption above) and i(t,0)=λ(t)S(t), Equation 15 reads as

(16) $$\frac{\text{d}I}{\text{d}t}=-\int_{0}^{\infty }\gamma (\tau )i(t,\tau )\text{d}\tau +\lambda (t)S(t)$$

It is reasonable to assume that the integral in Equation 16 has a finite upper bound ω<∞, because there are no infected people with infinite infection age τ. As i(t,τ)≥0, the Mean Value Theorem for Definite Integrals (9) guarantees the existence of $\tilde{\tau }=\tilde{\tau }(t)\in [0,\omega ]$ such that

(17) $$\frac{\text{d}I}{\text{d}t}=-\gamma (\tilde{\tau }(t))I(t)+\lambda (t)S(t)$$

Equation 17 is the same as Equation 13 with $r(t)=\gamma (\tilde{\tau }(t)),$ which gives an indication that Equation 3 from the infection-age-structured SIR model can indeed be approximated by Equation 13 from the conventional SIR model.

If it holds true that

(18) λ(t)S(t)=R(t)r(t)I(t)

we can reformulate Equation 13 with an explicit dependency on R. To see this, we assume that Equation 18 holds true and find

(19) $$\begin{array}{rl} \frac{\text{d}I(t)}{\text{d}t} & \overset{17,18}{=}-r(t)\;I(t)+R(t)r(t)I(t)\\ & =r(t)\;I(t)\;(R(t)-1) \end{array}$$

With the usual smoothness assumptions, Equation 19 has the unique solution

(20) $$I(t)=I(0)\;\exp\left(\int_{0}^{t}r(\sigma )\;\left[R(\sigma )-1\right]\text{d}\sigma \right)$$

where $I(0)=\int_{0}^{\infty }i_{0}(\tau )\text{d}\tau$ (note that $i_{0}$ was assumed to be integrable). Equation 19 (and equivalently Equation 20) directly relates the number of infected people I to the effective reproduction number R.

Now, we have to examine the conditions such that Equation 18 at least approximately holds true. As i(t,⋅) is non-negative, the Mean Value Theorem for Definite Integrals applied to the left-hand side of Equation 18 reads as

(21) $$\begin{array}{rl} S(t)\lambda (t) & \overset{5}{=}S(t)\int_{0}^{\infty }\beta (t,\tau )i(t,\tau )\text{d}\tau \\ & =S(t)\;\beta (t,\tau^{*}(t))\;I(t) \end{array}$$

for $\tau^{*}(t)\in [0,\omega ]$.

On the right-hand side of Equation 18, we have

(22) $$R(t)\;I(t)\;r(t)\overset{11}{=}S(t)\;\beta (t,\tau^{\prime }(t))\;I(t)\;r(t)\int_{0}^{\omega^{\prime }}\Gamma (\tau )\text{d}\tau$$

where we assumed that β(t,⋅)Γ has a compact support $\left[0,\omega^{\prime }\right]$ and $\tau^{\prime }(t)\in [0,\omega^{\prime }]$.

By comparing Equations 21 and 22, we see that $r(t)\int_{0}^{\omega^{\prime }}\Gamma (\tau )\text{d}\tau =1$ and $\beta (t,\tau^{*}(t))=\beta (t,\tau^{\prime }(t))$ imply the desired equality λS=RrI. Hence, if $r(t)=\gamma (\tau^{*}(t))$ is close to $(\int_{0}^{\omega^{\prime }}\Gamma (\tau )\text{d}\tau {)}^{-1}$ and $\beta (t,\tau^{*}(t))$ is close to $\beta (t,\tau^{\prime }(t))$, we can expect that Equation 20 is a reasonable approximation for the number of infected I(t) in the age-structured SIR model.

Equation 19 has the important advantage of being a linear ordinary differential equation with the analytic general solution Equation 20. Given the remission rate r(t) and the effective reproduction number R(t), the solution I(t) can be calculated at least numerically, for example, by Romberg integration (10). Given that r(t)>0, Equation 19 (or equivalently Equation 20) yields a very simple justification of the epidemiological commonplace that the number of infected people I is increasing over time t if and only if the effective reproduction number R(t) is greater than 1.

## 3 Simulation: lockdown during the SARS-CoV-2 pandemic

To demonstrate how good the approximation given by Equation 19 (or equivalently by Equation 20) is to describe the number of infected people I in the infection-age-structured SIR model, we use a simulation motivated from the SARS-CoV-2 pandemic. During the pandemic, many governments decided to invoke public health interventions to control the spread of the virus. Brinks et al. (6) simulated three consecutive periods of the epidemic in a hypothetical population. A phase of increasing number of infections from t=0 to 25 (days) is followed by a phase of implementation of a (strict) lockdown (from t=25 to 30). During the third phase (post-lockdown), the pandemic remains controlled (from t=30 to 60). The 5-day period following the start of the lockdown was chosen as the wash-in phase. The rationale for the wash-in phase is that public health interventions usually require some time before taking full effect (11). After this wash-in period, we assumed that the effect of the lockdown remains unaltered until the end of the simulation at day t=60.

The specific numbers for solving Equations 2–4 with initial Conditions 6–9 including their justifications are given in Brinks et al. (6). The supplement of Brinks et al. (6) also contains a description for the numerical solution of Equations 2–4 with initial Conditions 6–9 on a grid $(t_{m},\tau_{n})=(m\times \delta_{h},n\times \delta_{h}),m=0,\ldots ,M,n=0,\ldots ,N$, starting with $\left(t_{0},\tau_{0}\right)$, ending with $\left(t_{M},\tau_{n}\right)$, and equidistant step size $\delta_{h}>0.$ Apart from the setting of the simulation and its results, Brinks et al. (6) has a little overlap to the work presented here.

For the simulation done here, we calculate the incidence density i on the grid $(t_{m},\tau_{n})=(m\times \delta_{h},n\times \delta_{h}),m=0,\ldots ,M,n=0,\ldots ,N$, starting with $\left(t_{0},\tau_{0})=(0,0\right)$, ending with $\left(t_{M},\tau_{n})=(60,30\right)$, and equidistant step size $\delta_{h}=\frac{1}{24}$ (days). The transmission rate β(t,τ) is assumed to be the product of two functions $\beta (t,\tau )=\beta_{t}(t)\times \beta_{\tau }(\tau ).$ Note that the factorization into two factors is not a necessary condition and has been chosen for ease of representation, for details refer to Brinks et al. (6).

**Figure 1:**
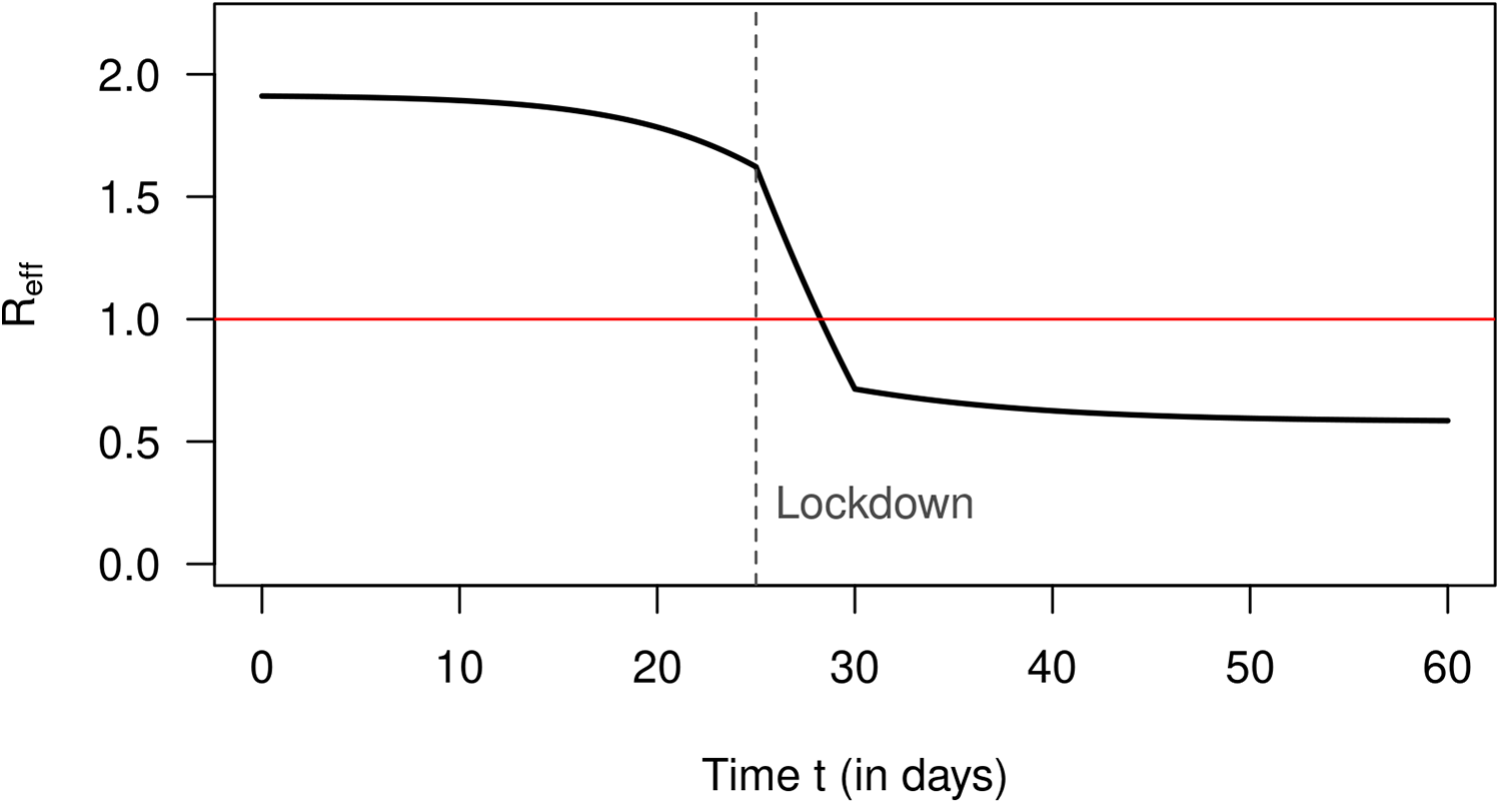
Reproduction number R (ordinate) over calendar time *t* (abscissa, in days) in the simulation [cf. Figure 5 in Supporting Information S1 of Brinks et al. (6)].

The removal rate γ is assumed to be constant $\gamma (\tau )=\frac{1}{4}.$ Applying Romberg integration (10) to Equation 11 yields the effective reproduction number R as depicted in Figure 1 [details can be found in Brinks et al. (6) and the associated supporting information S1]. We see that the lockdown reduces R quickly to values below 1, which is intended to control the spread of the disease. The steep decline of R is consistent with Fraser’s description of an abrupt switch from a high to a low value due to an effective intervention (5). Note that we do not consider the kind (or effectiveness) of the considered interventions. Here, it is important to simulate a realistic reduction of R (at least in magnitude).

**Figure 2:**
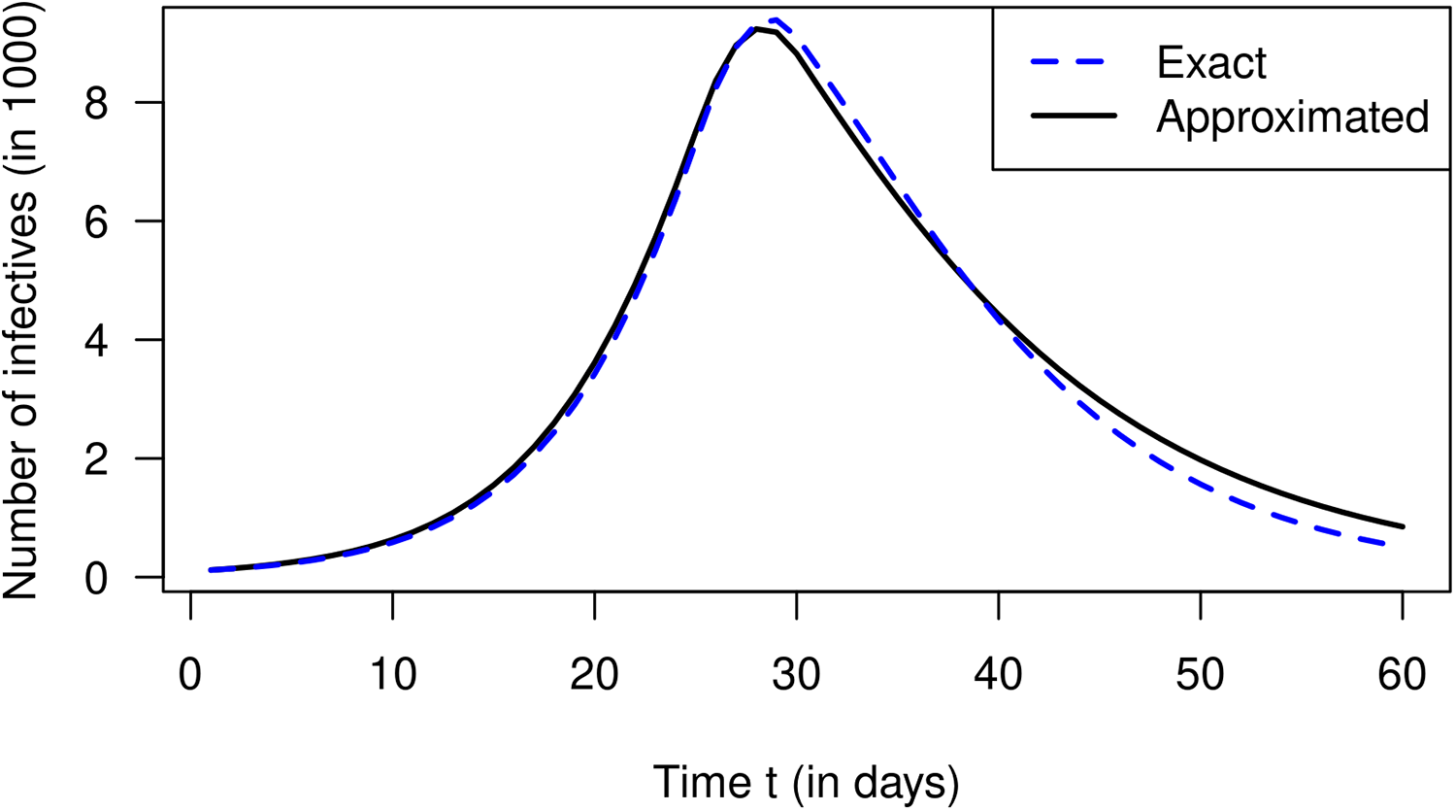
Number of infected people I over calendar time t in the simulation. The blue curve corresponds to the exact solution (Equation 1) while the black curve is the approximation via Equation 20.

So far, we have only used the theory of the infection-age-structured SIR model. To see if the approximation by Equation 20 (derived from the conventional SIR model) yields reasonable results, we solve Equation 20 with the calculated R values (as shown in Figure 1). With respect to the number of infected people I(t) over time t, we obtain the black graph as presented in Figure 2. For comparison, the exact I as calculated by Equation 1 (from the infection-age-structured SIR model) is shown as a blue curve. Periods of increasing and decreasing numbers of infected people coincide quite well in both curves. The approximated numbers (the black curve) deviate from the exact values (the blue curve) less than 10% between days 0 and 45. After day 45, when the number of infected people is already strongly decreasing, the approximated I values overestimate the true values considerably (up to 67% at day 60).

## 4 Application: pandemic influenza in Prussian army camps around Arnsberg (Germany)

**Figure 3:**
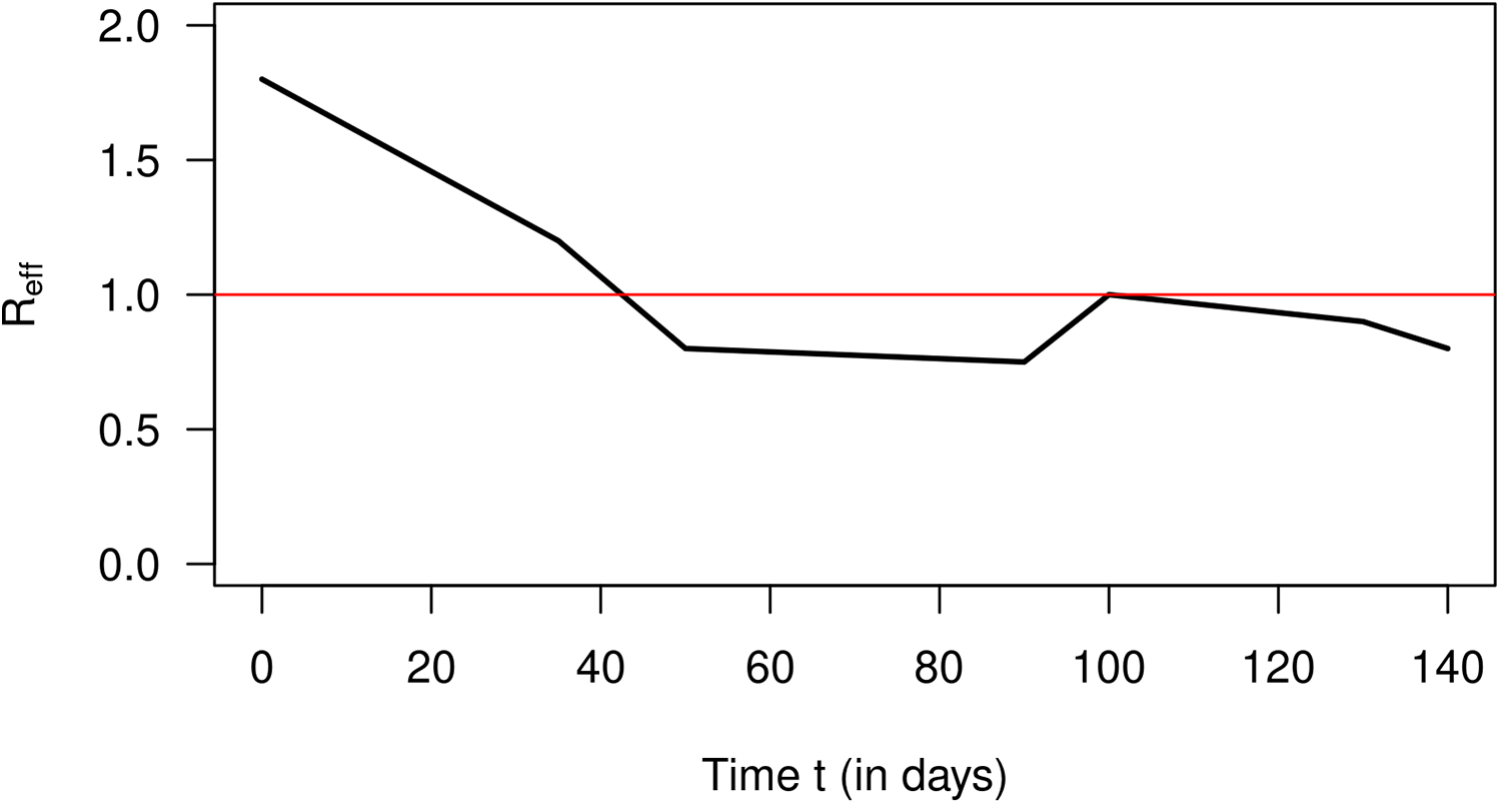
Approximate effective reproduction number R after 9 September 1918 (in days) for an influenza outbreak in Prussian army camps.

**Figure 4:**
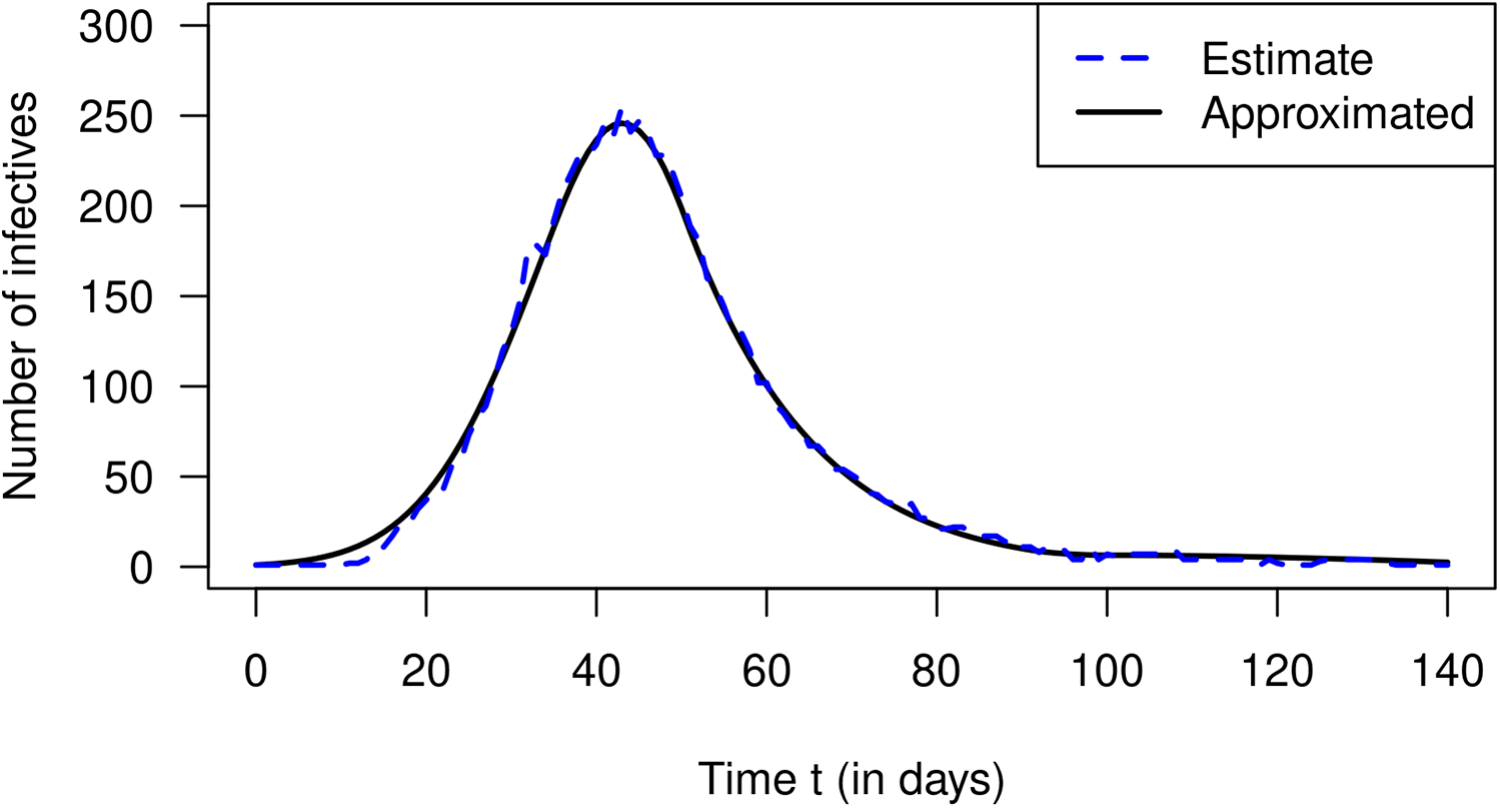
Number of infected people I (ordinate) over calendar time t in Prussian army camps in the region of Arnsberg (Germany). The blue curve corresponds to estimated numbers according to Nishiura (12) and the black curve is the approximation via Equation 20.

In 2007, Nishiura estimated the number of cases of incident influenza in Prussian army camps in the region of Arnsberg (Germany) from reported death cases during September 1918 and January 1919 (12). The estimated numbers of incident cases are extracted from Nishiura (12) and are published in Briggs et al. (13). Then, Nishiura and later Nishiura and Chowell estimated the effective reproduction numbers R from the case counts for a period of 140 days starting from 9 September 1918 (4, 12). The estimation method required the length of the generation time of the virus, which is unknown. To overcome the problem of the unknown generation time, Nishiura and Chowell used three scenarios with different generation times. We confine ourselves to a coarse schematic description of the temporal course of R shown in Figure 3. After the start of the outbreak, the effective reproduction decreases from about 1.8 to 0.7 on day 90, increases to 1 on day 100, and decreases after that again. Using these R values, we could reconstruct the estimated values of infected people by Equation 20. The result is shown in Figure 4. The black curve corresponds to the approximated I according to Equation 20 with constant r(t)=0.32. The number of estimated cases I is shown as a blue curve. As in the previous section about the simulation, the fit between the curves is reasonably well.

## 5 Discussion

In this article, we demonstrated that the number of infected people I in the infection-age-structured SIR model without demography of the background host can be approximated by a simpler differential equation based on the conventional SIR model. While the dependency on the effective reproduction number R in the infection-age-structured SIR model is implicit, the simpler differential equation makes the dependency explicit. This is advantageous for calculating I in situations when the effective reproduction number R is given, for example, by surveys or surveillance.

In a simulated example about a hypothetical lockdown during the SARS-CoV-2 pandemic, we could compare the exact number of infected people (calculated by the infection-age-structured SIR model) with the approximation during a 60-day period. Qualitatively, the two epidemic curves agree reasonably well over the whole simulated period. During the first 45 days, the absolute difference between the epidemic curves is below 10%. As the number of infected people decreases, the relative difference increases rapidly and reaches about 70% at the end of the 60-day period. Of course, this is also an effect of the relatively low absolute numbers of infected people at the end of the simulation.

Finally, we applied the approximation to real-world data from an influenza outbreak in Prussian army camps after the Second World War. We could compare the estimated number of infected people with the approximated number based on the R values. Again, the two epidemic curves qualitatively agree well.

In other prominent sources, the effective reproduction number R is defined via a renewal equation [see, e.g., Nishiura (12)] or as the spectral radius of the next generation operator (14). However, our approach is based on calculus and does not need these more sophisticated mathematical concepts. Thus, we believe that the important concept of the effective reproduction number might be accessible and useful to a broader audience.

During epidemic situations, the effective reproduction number R is frequently estimated from the number of reported cases (5). Recently, we have shown that this estimation is stable in case of incomplete case detection (6). At the moment, we do not have any indication that Equations 19 or 20 provide a better alternative for estimating R. The benefit of Equations 19 or 20 lies in the fact that an estimated R allows calculation of the true number of infected people I, which might be underestimated by incomplete case detection or under-reporting. The question whether to use Equation 19, or equivalently Equation 20, depends on the specific application and the preference of the user. We have chosen to use Romberg integration for Equation 20, because error estimates are easier to obtain than for numerical solutions to Equation 19 (10, p. 335).

To our knowledge, it is the first time that the dependency of I on the effective reproduction number R is made explicit by an approximation using a differential equation in the infection-age-structured SIR model. For the conventional SIR model, which is a special case of the infection-age-structured SIR model, a similar result has been found in Bettencourt and Ribeiro (15, Equation 2). For a recent study about approximating SARS-CoV-2 with the conventional SIR model, we refer the reader to Prodanov (16). Usually, the effective reproduction number R is defined in terms of the variables in Equations 2–4, like in Equation 11. In the literature, we frequently find the definition $R=R_{0}\;\times \;\frac{S}{S+I+R}$, which describes how R depends on the basic reproduction number $R_{0}$ and the variables S,I, and R defined above [see, e.g., Vynnycky and White (17)]. Conversely, Equations 19 and 20 describe how the number of infected I depends on R for the infection-age-structured SIR model. Hence, Equations 19 and 20 describe an opposite way than usual. In our work here, we could generalize the findings of Bettencourt and Ribeiro (15) for the conventional SIR model to the infection-age-structured SIR model. We note that the use of SIR models is not restricted to epidemiology but can also be used in examining the spread of rumors and news (18). With a view to news, the infection age refers to the time elapsed after a recipient got to know the new information.

By the differential Equation 19 and similarly Bettencourt and Ribeiro (15, Equation 2), the common interpretation of R as an indicator if the number of infected people I increases (R>1) or not is justified in a very simple and straightforward way. While the approximation works reasonably well in the examples about SARS-CoV-2 and influenza shown here, there might be diseases for which a higher accuracy is requested. The approximation of Equations 19 or 20 might not work well in all cases or other parameter constellations. To gain insight into these constellations, an extensive simulation study is necessary, which is beyond the scope of this article and is subject to future work. As long as error estimates are not known, careful consideration of the use of Equation 19 (or equivalently Equation 20) is necessary.

## Data Availability

The datasets presented in this study can be found in online repositories. The names of the repository/repositories and accession number(s) can be found in the article/Supplementary Material.
